# Learning Needs of Patients with Cancer and a Pre-Existing Autoimmune Disease Who Are Candidates to Receive Immune Checkpoint Inhibitors

**DOI:** 10.3390/cancers15154004

**Published:** 2023-08-07

**Authors:** Maria A. Lopez-Olivo, Johncy J. Kachira, Maryam Buni, Sang Taek Kim, Huifang Lu, Jean H. Tayar, Gabrielle F. Duhon, Juan I. Ruiz, Clifton O. Bingham, Cassandra Calabrese, Robert J. Volk, Maria E. Suarez-Almazor

**Affiliations:** 1 Department of Health Services Research, The University of Texas MD Anderson Cancer Center, Houston, TX 77030, USA; johncyjohn6861@gmail.com (J.J.K.); gfduhon@mdanderson.org (G.F.D.); jiruiz1@mdanderson.org (J.I.R.); bvolk@mdanderson.org (R.J.V.); msalmazor@mdanderson.org (M.E.S.-A.); 2 Section of Rheumatology and Immunology, Department of General Internal Medicine, The University of Texas MD Anderson Cancer Center, Houston, TX 77030, USA; mbuni@mdanderson.org (M.B.); kst7766@hotmail.com (S.T.K.); hlu@mdanderson.org (H.L.); jhtayar@mdanderson.org (J.H.T.); 3 Division of Rheumatology, Department of Medicine, Johns Hopkins University, Baltimore, MD 21224, USA; cbingha2@jhmi.edu; 4 Cleveland Clinic Foundation, Cleveland Heights, OH 44195, USA; calabrc@ccf.org

**Keywords:** qualitative interviews, patient education, immunotherapy, autoimmune diseases

## Abstract

**Simple Summary:**

Our results can be used in clinical settings to facilitate patient–provider discussion. They can also be used to develop educational interventions to increase knowledge and decrease uncertainty. We identified seven key informational needs: (1) possible adverse events; (2) benefits of ICI; (3) ICI mechanism of action in the context of autoimmune disease; (4) management of flare-ups while receiving ICI; (5) reasons for stopping or modifying cancer or autoimmune disease treatment; (6) likelihood of autoimmune disease progression or organ damage; and (7) lifestyle changes that could help avoid adverse events.

**Abstract:**

Patients with pre-existing autoimmune disorders and cancer considering immune checkpoint inhibitors (ICIs) need to receive balanced information about the benefits and risk of developing immune-related adverse events (irAEs) and flare-ups of their autoimmune disease. To assess the learning needs of patients with cancer and pre-existing autoimmune disease regarding ICI treatment, we interviewed 29 patients with autoimmune disease and cancer from a comprehensive cancer center, of whom 20 had received ICI and 9 were candidates to receive ICI at a US Cancer Center. In-depth semi-structured interviews were conducted from August 2021 and January 2022. Interviewee’s opinions and preferences about content and information delivery methods were collected. We recorded and transcribed interviews and analyzed them using thematic analysis. Half of the participants were female, and their median (SD) age was 62.9 (±10.9) years. The identified health information needs included the following: (1) information on irAEs and autoimmune disease flare-ups; (2) benefits of ICI; (3) ICI mechanism in the context of autoimmune disease; (4) management of flare-ups; (5) reasons for stopping or modifying cancer or autoimmune disease treatment; (6) likelihood of autoimmune disease progression or organ damage; and (7) lifestyle changes that could help avoid irAEs. Patients who had received ICI and those who had not yet received treatment reported similar needs, although patients who had received ICI had more questions about cancer treatment modifications. Patients also expressed the need to better understand when to contact their provider and how to share information with multiple providers. Most patients wanted to receive information in visual formats for review at home and at their own pace. Patients expressed interest in having educational tools to facilitate shared decision-making with their physicians, and they identified several areas of health information concerning therapy with ICI. They also highlighted the importance of communication among their various providers.

## 1. Introduction

Depending on the type of cancer, pre-existing autoimmune diseases could be present in a substantial percentage of patients. For example, in a real-world analysis, up to 25% of patients with lung cancer also had an autoimmune disease [1]. In patients with both cancer and pre-existing autoimmune disease, cancer can be managed with immune checkpoint inhibitors (ICIs). Tumor responses are comparable to those in patients without autoimmune disorders, though the data conflict regarding the type and severity of immune-related adverse events (irAEs) in patients with autoimmune disease compared to patients without these conditions [2,3]. However, up to 50% of patients with autoimmune disease experience an exacerbation of their autoimmune condition while on ICI treatment [4,5,6,7].

Information on the benefits and risks of ICI is critical for patients who have both an autoimmune disease and cancer and who are candidates for ICI treatment. Patient education can facilitate patient–provider communication, improve the tracking and reporting of adverse events, and help patients to feel more involved in and satisfied with their care [8,9]. However, educational resources tailored to the informational needs of this patient population have not yet been developed. Therefore, we assessed the educational content and information delivery methods that could enhance patient–provider communication regarding the potential use of ICI treatment in patients with pre-existing autoimmune disease.

## 2. Materials and Methods

The reporting of this study followed the Standards for Reporting Qualitative Research [10].

### 2.1. Qualitative Approach and Research Paradigm

This qualitative, interview-based study used a grounded theory approach in which data collection and analysis were not tested against any hypotheses; our analysis was thus focused on answering our research questions rather than applying theoretical concepts. Our goal was to gain insight into the health information these patients need when discussing ICI treatment with their providers. We followed a constructivist paradigm because the participants’ views were directly influenced by their own experiences [11].

### 2.2. Researcher Characteristics and Reflexivity

Interviews were led by trained research staff (G.D. or A.N.) who encouraged open discussion. The interviewers had no relationship with study participants prior to the interviews. Additional members of the research team were doctoral-level experts in qualitative research (R.J.V., M.E.S.-A., M.A.L.-O., J.J.K.), knowledge synthesis (M.A.L.-O., M.E.S.-A.), patient education (R.J.V., M.E.S.-A., M.A.L.-O.), internal medicine (J.I.R.), and rheumatology (M.E.S.-A., C.O.B., C.C., M.B., S.T.K., H.L. J.T.).

### 2.3. Context and Sampling Strategy

We identified potential participants from clinic schedules and evaluated their eligibility through a chart review from August 2021 to August 2022. Included patients (1) had a diagnosis of cancer; (2) had a diagnosis of pre-existing autoimmune disease (Supplementary Table S1); (3) were aged 18 years or older; (4) were candidates for treatment with any currently available ICI (according to treating clinicians) or were patients receiving, or who had already received, ICIs and had already made treatment decisions (this allowed us to capture the needs and preferences of patients who had already experienced the decision and understood what it entails); (5) had telephone or email access; and (6) were able to communicate in English without a translator. We used convenience sampling to enroll 30 patients. This sampling technique was chosen owing to the limited number of patients who met the inclusion criteria and agreed to participate in the study. The study team considered the sample size and composition to be appropriate for achieving data saturation and determining the learning needs of the target population.

### 2.4. Ethical Issues Pertaining to Human Subjects

The study protocol was reviewed and approved by the Institutional Review Board of the University of Texas MD Anderson Cancer Center (protocol # 2020-0035). Verbal consent was obtained from all participants before interviews. Data were securely stored as per institutional guidelines.

### 2.5. Data Collection Methods

Eligible patients were invited to participate in the study. In-depth semi-structured interviews were conducted over the phone between August 2021 and January 2022. Interview times ranged from 25 to 45 min, and the audio was recorded. We also collected patient demographic data (age, sex, language, marital status, education level, ethnicity, and employment status), date of autoimmune disease onset, autoimmune disease medications, and cancer type and stage.

### 2.6. Data Collection Instruments and Technologies

The interview guide was developed by the research team (Supplementary Table S2). We adapted the interviews to accommodate the different perspectives of patients at different points in the continuum of care. Participants who had already received immunotherapy were asked what information they considered important to convey so that patients with an autoimmune disease considering ICI treatment would feel better informed.

### 2.7. Data Processing

Interview recordings were transcribed verbatim. Transcripts were anonymized using identification codes. A team member (H.D.) conducted a line-by-line check of the accuracy of the transcripts. The verified transcripts were imported into Dedoose version 9.0.17 for data coding and analysis [12].

### 2.8. Data Analysis

Demographic data were descriptively analyzed. The steps described by Braun and Clarke [13] were used to guide the thematic analysis. First, the PI (M.A.L-O.) independently read the transcripts to become familiar with the data. Then, 2 researchers (J.J.K. and H.D.) independently performed initial coding of the data. We classified the codes into pre-themes and reviewed multiple iterations of the pre-themes until agreement was reached. The pre-themes were then compiled into themes and subthemes. We defined and labeled the themes/subthemes and confirmed that they corresponded with the codes and the data. Finally, we selected quotes that best represented each theme and subtheme.

### 2.9. Techniques to Enhance Trustworthiness

The first 2 authors and H.D. collaborated on coding decisions (triangulation) to enhance credibility [14]. Dependability was established through prolonged engagement, reflection on previous research experience, understanding of the topic of study, and expert checking [15]. Transferability was upheld with an audit trail. For confirmability, 2 interviews were selected for a non-causal random institutional audit, and all standards for research without bias were met.

## 3. Results

### 3.1. Participants

Of the 29 interviewed patients, 15 (50%) were female, and their median (SD) age was 61.9 (±15.5) years (Table 1). The most common autoimmune disease reported was rheumatoid arthritis. Most (70%) of the patients had melanoma. At the time of interview, 66% had completed or discontinued ICI treatment.

### 3.2. Synthesis and Interpretation of the Data

**Figure 1:**
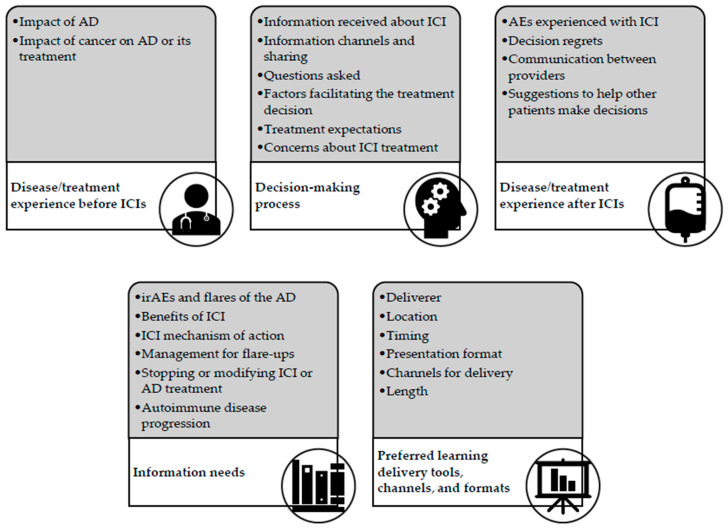
Themes and subthemes identified in the interviews. Abbreviations: ICI, immune checkpoint inhibition; AE, adverse event; AD, autoimmune disease; irAE, immune-related adverse event.

Out of 2092 coded passages, we identified five themes that addressed our research objectives: disease/treatment experience before ICIs; factors related to decision making; disease/treatment experience after ICIs; key information needs; and preferences for delivery of information on ICIs (Figure 1).

#### 3.2.1. Disease/Treatment Experience before ICIs

Patients were asked what aspects of their lives were most affected at the time they made the decision to start ICI treatment. We categorized their responses into two subthemes.

1. Impact of autoimmune disease: Only a few participants felt their autoimmune disease was well controlled before they started ICI treatment. Among patients with uncontrolled autoimmune disease, multiple areas of mental (e.g., cognitive, stress), physical (e.g., fatigue, pain), and social (e.g., personal relations, financial challenges) well-being were affected due to their autoimmune disease.
2. Impact of cancer on autoimmune disease or its treatment: Although some participants were advised to stop or modify their autoimmune disease treatment after their cancer diagnosis, leading to substantial exacerbation of autoimmune symptoms, patients agreed that treatment of the cancer should be prioritized. In other cases, the cancer did not affect patients’ autoimmune disease, or cancer treatment even improved the symptoms of the autoimmune disease.

#### 3.2.2. Decision-Making Process

This theme encompassed six subthemes related to the information that had been communicated to the patient and the factors that facilitated their decision, including their expectations and concerns.

1. Information about ICI: Most patients received explanations of their cancer, the mechanism of action of ICIs, probable treatment outcomes, the possibility of autoimmune disease flare-ups and irAEs, warning symptoms to be aware of, and indicators of non-response. However, a few patients felt that their oncologist was not aware of all potential autoimmune disease complications and felt better informed by their autoimmune disease specialists.
2. Information channels and how patients shared information: All participants first learned about ICIs, including their benefits and risks, from their oncologists. Some patients went to Google or the drug manufacturer website to find information about potential adverse effects of and current research on ICIs. Others went to social media to learn about others’ experiences with ICI treatment. A few people shared the information they learned with their providers.
3. Questions asked: Patients who had already received ICIs listed several types of questions that they had asked their medical teams: questions about the probability of treatment success for cancer in their individual case, specific adverse effects of concern, and the expected duration of treatment. Patients who had not received ICIs were less likely to provide specific questions. A few participants explained that they had not yet developed questions because they were waiting to see their providers to learn about test results, while others had questions regarding their specific treatments, such as the concomitant use of chemotherapy and potential implications.
4. Factors facilitating the treatment decision.: Most participants reported that the decision to start ICI treatment had been made by their oncologists. However, participants with stage III resectable melanoma stated that the decision to start ICI was shared by patient and provider. Participants reported having based their decisions on the information provided by their providers, the probability of survival, their confidence in their oncology team, their satisfaction with the responses to their questions, and the reputation of the institution. None of the participants considered their autoimmune disease a factor in their decision to start ICI treatment. Some participants wanted to start ICI treatment as quickly as possible because they had exhausted other cancer treatment options, or their expected survival was limited without ICI.
5. Treatment expectations: Most patients felt that ICI treatment could increase the probability of survival by inducing either long-time remission or cure; a few patients expected that ICIs would only relieve their pain or decrease the tumor size. Similarly, although patients received information about adverse effects and the possibility of autoimmune disease flare-ups, more than half of the patients expected few to no adverse effects or flare-ups. Furthermore, based on their experience with chemotherapy, a few patients thought that ICI treatment could help with their autoimmune disease.
6. Concerns about ICI treatment: A few participants perceived ICI to be safer than chemotherapy. Most patients expressed concerns about adverse effects and how adverse events would impact efficacy of ICI. Regarding adverse effects, participants were most often concerned about the probability of flare-ups or chronic complications that could affect their quality of life, e.g., patients did not want to be bed-ridden or lose their independence or ability to have a normal life. Some were concerned about the potential treatment for flare-ups of their autoimmune disease or adverse effects and its impact on the ICI’s efficacy or the need to pause or discontinue the ICI. Regarding efficacy, some patients expressed fear of leaving people behind or of their deaths being a burden for their family.

#### 3.2.3. Disease/Treatment Experience after ICIs

Patients who had received ICI treatment were asked if they had experienced flare-ups or adverse effects, their thoughts about their decision to initiate ICI treatment, and the type of information they deemed important to help other patients. Their responses were categorized into four subthemes.

1. irAEs and flare-ups experienced with ICI: Half of the 20 participants who had received ICI treatment reported experiencing an exacerbation of their autoimmune disease, with 5 (25%) reporting both irAEs and a flare; irAEs included rashes, colitis, and other gastrointestinal issues. Flare-ups of autoimmune disease were most often described as severe, leading to a change in treatment for their autoimmune disease or cancer, and, in a few cases, hospitalization.
2. Decision regrets: Most participants reasoned that given their limited options, they wanted to do everything possible to avoid cancer progression. Patients who had no irAEs expressed no regrets about ICI treatment. Of those who experienced irAEs, all but two agreed that being alive was worth the flare-ups or irAEs; those two patients expressed mixed feelings about ICI treatment because irAEs had caused long-term complications or permanent damage (i.e., neuro-inflammation, retinal degeneration).
3. Communication between oncologist and autoimmune disease specialist: Most patients were under the impression that their oncologist and autoimmune disease specialist maintained regular communication about their treatment and progress. Some patients had concerns that one or more clinicians had been uninformed or had not communicated with others. Patients reported sending medical records to each of their clinicians to ensure all had the same information.
4. Suggestions to help other patients make decisions: Most patients highlighted the importance of trusting their medical teams. They recommended that future patients understand the benefits and risks not only of ICI treatment but also its alternatives, take time to process information, improve their overall health and control of their autoimmune disease, and have confidence in asking questions. They also suggested an emergency line for on-demand answers or monthly check-ups.

#### 3.2.4. Information Needs

This theme encompassed seven subthemes related to the information patients with pre-existing autoimmune disease thought was most important to include with respect to educational content for similar patients.

1. Information on irAEs and flare-ups of the autoimmune condition: All patients agreed on the importance of information about the probabilities of having irAEs and/or flare-ups. Most patients deemed it to be of key importance to have access to a list of common and concerning symptoms when receiving ICI. A few patients also wanted information about the probability of chronic or fatal irAEs. Many participants had requested information on how best to monitor their symptoms and reach out to their medical team.
2. Benefits of ICI and general information about ICI treatment: Patients perceived survival rates to be an important factor in comparing treatment options, including the option to decline ICI treatment. They wanted to understand the effectiveness of ICIs compared to other options and whether ICIs were equally effective for all cancer stages. Patients also wanted to receive guidance in monitoring the effects of treatment (e.g., how to know if it was working) and to have a clear understanding of what successful treatment would look like. Some patients stated that they would use the information to decide if their personal affairs needed to be put in order. General information about the process for receiving ICIs (e.g., laboratory tests and referrals needed; follow-ups) was deemed important by some patients. A few patients wanted to learn whether the ICI would be given alone or with other non-ICI treatments. Patients also wanted to receive information about the differences among ICI agents.
3. ICI mechanism of action in the context of autoimmune disease: Patients wanted to better understand how ICIs work in people with autoimmune disease, specifically the implications of “supercharging” an immune system that is already overactive. Some patients also wanted to know if having more than one autoimmune disease would result in more irAEs or more severe complications.
4. Management for flare-ups: Most patients wanted to learn about the expected severity of potential flare-ups for people with similar clinical characteristics, how long flare-ups would last, and the expected frequency of flare-ups during ICI treatment. Patients also expressed the importance of understanding the role of steroids in managing flare-ups, how steroid use impacts the efficacy of ICIs, and their insurance coverage in case of flare-ups.
5. Possible reasons for stopping or modifying treatment (for cancer or autoimmune disease): Some of the interviewed patients expressed interest in receiving information on disease progression to better understand possible reasons for changing cancer or autoimmune disease treatment plans. Similarly, some participants mentioned the importance of knowing, on average, how long people could receive ICI before experiencing a flare-up. Some participants also wondered what to expect after discontinuation of the ICI.
6. Likelihood of autoimmune disease progression or organ damage: Patients wanted to learn the best time to start ICI treatment according to their autoimmune disease status (for instance, whether they could start ICI treatment as soon as possible regardless of whether their autoimmune disease was controlled or uncontrolled). They wanted to know if they should expect symptoms that could hinder their daily activities, such as cognitive issues, fatigue, or inability to exercise. In addition, most participants wanted to understand the potential ICI-related complications of their autoimmune disease; for example, mobility loss, deformities, joint fusion, pain, or organ damage. Participants who had already received ICI emphasized the importance of having specialists conduct a baseline evaluation of the autoimmune disease before initiating ICI treatment so that disease progression could be tracked.
7. Lifestyle changes to help avoid irAEs: Patients also wanted to receive information on how to mitigate irAEs; for instance, via lifestyle changes such as diet modification, exercise, probiotics or supplements, and alcohol and smoking cessation.

#### 3.2.5. Preferred Learning Delivery Tools, Channels, and Formats

1. Preferred person to deliver the information: Most people preferred to receive information about ICI treatment directly from their providers. However, a few wanted to learn on their own, have the opportunity to digest the information, and ask questions later. Three patients preferred to receive information from and to ask questions of health educators or nurses, focusing their medical encounters only on discussing concerns.
2. Preferred location: Most patients wanted to receive information during a provider visit or in the clinic/hospital setting. Others preferred to receive information in a quiet place such as their home or a patient learning media center. Many preferred to have access to the information at any place and time so that they could access it when they felt comfortable, pause their progress through the information, or repeat information as needed.
3. Preferred timing for receiving information: Most patients wanted to receive educational resources before meeting with the provider to ensure that they had time to go over the material, discuss it with significant others, and formulate questions or points to discuss during the visit. Others preferred to receive information during their provider visit, then review the information and ask questions later. A few patients preferred to receive information multiple times (e.g., online before the provider visit (online material), discussion during (patient–doctor discussion) the visit, and review after the visit (through discussion with pharmacist)).
4. Preferred presentation format: Many patients commented on design aspects of educational materials that would optimize understanding. Patients mostly preferred visual presentations, but a few favored audios. Patients commonly preferred simple and concise presentations of data through drawings or basic graphics/charts. A few patients suggested incorporating testimonials to help convey detailed or complex data or to create a better connection with the reader. Patients felt that learning about others’ struggles with the same health issues and the same treatment was more helpful than looking at numbers.
5. Preferred channels for delivery: The most common delivery channels suggested were via website and mobile app. Websites and apps would support educational materials in multiple formats, such as video, audio, graphics, and text, and of various types; for example, patients suggested providing a list of concerning adverse events or an individualized risk calculator for probabilities of irAEs/flare-ups. In addition, patients suggested that a website or app could be integrated with the electronic health record or incorporate interactive features such as medication reminders. Other preferred formats were videos (which patients suggested could be narrated by patients and/or providers, enable viewers to select their preferred language, feature pictures and animations, and have a positive, inspirational framing), printed materials (e.g., handouts including graphics and summaries of key information), an online patient portal linked to the EMR, in-person conversations and/or classes, and emails.
6. Preferred length: Some patients preferred shorter educational materials, though a wide range of durations were mentioned (e.g., minimum 3 min, up to 25 min, 1–2 pages). Other patients felt better informed when they received longer material that included detailed information (e.g., minimum 30 min, 8–10 pages) but requested the inclusion of a short summary page at the beginning of the material.

### 3.3. Differences According to ICI Exposure

Patients who had received ICI treatment and those who had not yet received ICI treatment reported similar needs, but patients who had received ICI treatment had more questions about cancer treatment modifications (Table 2).

## 4. Discussion

We assessed the needs and preferences of cancer patients with autoimmune diseases who had received or were candidates to receive ICIs to understand the learning content and delivery methods that could enhance shared decision-making about ICI treatment given the increased risk of disease flare-ups these patients face when receiving ICIs [5,6,7]. Currently, no educational resources are tailored to the information needs of this population [16]. Our study provides the information needs of these patients and their preferences for the delivery of this information. 

Our interviews identified seven key information needs among these patients: likelihood of irAEs or flare-ups; expectations of success with ICIs; how ICIs work in the context of pre-existing autoimmune diseases; flare-up management; cancer or autoimmune disease treatment modification and discontinuation; likelihood of long-term harm; and lifestyle changes that could ameliorate potential harms. No prior studies have identified these key needs. Earlier qualitative and mixed-methods studies ascertained the perspectives of patients with cancer but not those with pre-existing autoimmune conditions [17,18,19,20,21,22,23,24,25,26,27,28]. For instance, Fraterman et al. [17] found that patients without pre-existing autoimmune disease receiving ICIs preferred customizable educational tools and notifications. As in our study, participants in that study desired information on management of irAEs, supportive care services, physical and mental well-being, symptom monitoring, and communicating with providers [17]. Similarly, Kamminga et al. [18] found that cancer survivors who had received ICIs deemed it important to learn how to communicate with their providers. Another study reported that quality of life influenced the decision to continue receiving ICI treatment among patients with ICI-induced inflammatory arthritis [21]. Although patients in both studies valued preservation of life over potential adverse events, participants in our study also expressed concerns about long-term complications that would affect their quality of life. Another topic covered in prior mixed-methods studies is the necessity of raising patients’ awareness of risks associated with ICIs given that patients often expect the severity of ICI-associated adverse events to be lower than that of non-ICI cancer treatments such as chemotherapy [19,20].

Interestingly, some evidence suggests that physicians and patients may have different perspectives on patients’ information needs. In a qualitative study reporting the determinants of ICI prescription and in our previous study evaluating physicians’ views on information needs in this population, clinicians perceived general information about ICI treatment to be more useful than personalized or specific information [29,30]. In contrast, the patients who participated in our study expressed the need for more detailed information and personalized risk/benefit probabilities. We observed several additional differences from our previous study involving physicians. Although the key information needs raised by physicians were similar to those raised by patients, several were raised only by patients: reasons for stopping or modifying treatment (for cancer or autoimmune disease); when to contact the provider; the possibility of autoimmune disease progression or organ damage; the need for sharing information among providers; and lifestyle changes that may ameliorate adverse effects [30].

Our study has some limitations. Our findings are derived from a single U.S. comprehensive cancer center; the educational needs and preferences of patients being treated at other types of centers or in other countries may differ. Moreover, subanalyses based on the type of autoimmune disease were not possible due to the limited number of participants per disease, and although we included patients with common autoimmune diseases, not all autoimmune diseases could be represented. Patients with rarer autoimmune diseases may have different perceptions about the educational material they need. Nevertheless, further interviews are unlikely to alter our findings given that we employed a semi-structured interview format with open-ended questions and reached thematic saturation after 16 patients. Additionally, as in all qualitative research, the observations, goals, and biases of the researchers may have affected the outcomes. We made an effort to reduce these biases by assessing the data without making any prior assumptions and solely using the everyday language of the interviewees in our analyses. 

## 5. Conclusions

In conclusion, we learned from patients about their experiences before and/or after receiving ICIs, their participation in the treatment decision-making process, the educational content that they thought could help future patients, and their preferred learning delivery tools, channels, and formats. Our results will be used to develop and test the acceptability and feasibility of an educational intervention to increase knowledge and decrease decisional conflict in cancer patients with pre-existing autoimmune disease who are considering ICI treatment. They can also be used in clinical settings to facilitate patient–doctor discussion.

## Figures and Tables

**Table 1:** Characteristics of the participants (N = 29).

Characteristic	No. (%) ^1^
Age, years, mean (±SD)	61.9 (±15.5)
Sex, Female	14 (48.3%)
Marital status	
Married/living with a significant other	24 (82.8%)
Divorced/Widowed	5 (17.2%)
Education level	
High School diploma or equivalent	4 (13.8%)
Some college/Bachelor’s degree/Associate’s degree	20 (69.0%)
Master’s degree/Advanced degree	5 (17.2%)
Race	
Non-Hispanic White	26 (89.7%)
Non-Hispanic Black/African American	3 (10.3%)
Have received ICI	20 (69.0%)
Type of autoimmune disease	
Rheumatoid arthritis	14 (48.3%)
Psoriasis	6 (20.7%)
Inflammatory bowel disease	3 (10.3%)
Psoriatic arthritis	3 (10.3%)
Ankylosing spondylitis	2 (6.9%)
Systemic lupus erythematosus	1 (3.4%)
Active autoimmune disease at the time of starting ICI	15 (50%)
Type of cancer	
Melanoma	21 (72.4%)
Lung cancer	4 (13.8%)
Renal/bladder carcinoma	4 (13.8%)

^1^ Unless otherwise specified. ICI, immune checkpoint inhibitor.

**Table 2:** Example quotes for salient subthemes.

Subtheme	Patient Who Received ICI	Patient Who Had Not Yet Received ICI
Disease/Treatment experience before ICIs		
Impact of autoimmune disease	*“Okay. It would be just basically daily living skills: cleaning, exercising, babysitting the grandkids.”*	*“ … I was constantly having joint pain, like seven-eight level joint pain.”*
Impact of the cancer on autoimmune disease or its treatment	*“… I think I’d rather be in a wheelchair than be dead, okay”*	*“… I don’t think it’s affected it any more than what it was when I had it before.”*
Decision-making process		
Information received about ICI	*“Nivolumab may have been a new enough medication that even my doctor, my oncologist, wasn’t fully aware of its risks…”*	*“I mean, they were pretty thorough in providing me with information about the side effects of the drug …”*
Information channels and how patients shared the information	*“… I would go online, and I would read everything that I could find about it. And then eventually, I actually jumped into some social media groups and saw what different people were experiencing.”*	*“I don’t—you know, I guess, you know, we look on a Facebook page of people all across the world that deal with this type of cancer …”*
Questions asked	*“… [My doctor] already had addressed those [questions] in describing how the treatment was and all the stuff I’d be going through.”*	*“Well, there’s nothing much I can ask him until those test results come back. Then I’m sure I’ll have a million questions …”*
Factors facilitating the treatment decision	*“[The doctor] explained so well that I just said, okay, you’re right. We need to do this.”*	*“My confidence in [INSTITUTION] and being impressed with the oncologist I was assigned to …”*
Treatment expectations	*“Hopefully, they’ll reduce my tumors. I’ll be cured, or at least to a point where my cancer is manageable.”*	*“… I understand that chemo is not very effective and that immunotherapy has over the last five years made a lot of changes in terms of life survival.”*
Concerns about ICI treatment	*“Probably the pain first and then the fatigue … And, of course, dying, you know, nobody wants to die …”*	*“[None] At this point, [chemotherapy] is doing more damage than good … Yeah, if there’s an alternative, I’m going for it.”*
Disease/Treatment experience after ICIs (or anticipation of effects for patients who had not received ICIs)		
irAEs and flare-ups experienced with ICI	*“… I couldn’t roll over and get out of bed. And so it was about three days max before I was affected, arthritis was affected.”*	*“I also asked my doctor … the options of stopping [ICI] and switching to something else because you never really know like how it’s going to really affect your body until you take it.”*
Decision regrets	*“… I don’t know that it was nivolumab in particular or just immunotherapy in general that caused my side effects that I am dealing with for the rest of my life. So, I couldn’t really say.”*	*“… I just ultimately wanted to make sure that I was doing everything I could to keep that from getting progressively worse even though there might have been a little bit of a chance that I would have some of the autoimmune symptoms …”*
Communication between oncologist and autoimmune disease specialist	*“I just—whenever I go to meet with them for my appointment, I will let them know whatever. That hey, I met with Dr. So-and-so—*	*“Make sure that they can get those medical records from that other physician so that they have that as well …”*
Suggestions to help other patients make decisions	*“Probably just overall health; where they’re at in their health and whatnot.”*	*“… to trust your doctors and to trust that medication and the research that’s been done on it …”*
Information needs		
Information on irAEs and flare-ups of the autoimmune condition	*“… information about whether or not the nivolumab would aggravate your rheumatoid arthritis”*	*“Side effects, you know, basically, you know, just the side effects and how long the course …”*
Benefits of ICI and general information about ICI treatment	*“—the long-term history of success of the nivolumab for treating melanoma.”*	*“I would have looked at the specifics of survival rates if given to me.”*
ICI mechanism of action in the context of the autoimmune disease	*“When they said it supercharged the immune system, I thought, isn’t that kind of what arthritis is already? … Is this going to cause a problem?”*	*“… I’m not real clear exactly how it works … The more I read about it, the more confused I get.”*
Management for flare-ups	*“… what the treatment plan options will be if [flare-ups] happens, so that way there’s already a game plan kind of set up.”*	*“…what are the medications that I need to have on hand or who I will be able to get in touch with if I have any side effects.”*
Possible reasons for stopping or modifying treatment (for cancer or autoimmune disease)	*“… I also asked my doctor about the possibility of restarting the treatment and if it ended up being something that … really make me feel real horrible or whatnot, the options of stopping [ICI] and switching to something else …”*	*“… I’ll ask … when are they seeing that there could be progress or not progress? How many treatments will it take to find out … How long will they wait to see before they want to possibly change their plan?”*
Likelihood of autoimmune disease progression or organ damage	*“I think I would have liked to have known more about the fatigue and cognitive issues …”*	*“… Am I going to have to take off work because … I work, especially to pay the bills …”*
Lifestyle changes that can be incorporated to help avoiding irAEs	*“… I read all these books on autoimmune disease and … I changed my diet, and I think that helped more than anything”*	*“I think if you display the benefits as well as the negative consequences out front … And lifestyle changes …”*
Preferred learning delivery tools, channels, and formats		
Preferred person to deliver the information	*“Oh, I could learn on my own, and if I have questions, I’ll call them, …”*	*“Health educator or professional of some sort. It alleviates some of the confusion because you have a real person right there …”*
Preferred location	*“… other than just the doctor and speaking with them in the office, definitely research at home just because it’s a place you’re familiar with …”*	*“I think at home. Just about everybody has a computer, and they can access this website and sit there at their leisure …”*
Preferred timing to receive the information	*“Before they start their treatment just so that we see the broader picture.”*	*“… probably after the visit … to review the information.”*
Preferred presentation format	*“… like a little extension to MyChart or something, I’d just sort of keep track of side effects … and be able to kind of communicate …”*	*“…a website … If I had an interactive format, you learn as you make a mistake, as long as it directs you back and explains …”*
Preferred channels for delivery	*“I like to have things in front of me that I can just pull out when I have time …I have my phone with me all the time.”*	*“… something that I can read followed by conversation.”*
Preferred length	*“Probably with more detailed information about what all has occurred. But I would think the main points …no more than two pages.”*	*“… Like if [people] really want to get down to dig deep into the details, they can do that. But to have a summary.”*

## Data Availability

The data that support the findings of this study are available from the corresponding author, [M.A.L.-O.], upon reasonable request. The data are not publicly available due to containing information that could compromise the privacy of research participants.

## Supplementary Materials

The following supporting information can be downloaded at: https://www.mdpi.com/article/10.3390/cancers15154004/s1, Table S1: eligibility criteria for the autoimmune disease of interest. Table S2: Semi-structured interview used with our participants.
