# Supplementary material for: Learning Needs of Patients with Cancer and a Pre-Existing Autoimmune Disease Who Are Candidates to Receive Immune Checkpoint Inhibitors
Source: Cancers (Basel). 2023 Aug 7;15(15):4004. doi: 10.3390/cancers15154004 (PMC10416973; doi:10.3390/cancers15154004)
Supplement: Supplementary file 1 [file cancers-15-04004-s001.zip › cancers-2527582-supplementary.pdf]

Supplementary Table S1. Eligibility criteria for the autoimmune disease of interest.

Disease of Interest	Criteria
Inflammatory bowel disease	1) Diagnosis of Crohn disease or ulcerative colitis by a gastroenterologist<br>2) For ulcerative colitis, proof of chronic changes over time (i.e., 6 months) and signs of inflammation histologically<br>3) Treatment with aminosalicylates, corticosteroids, thiopurines, or immune modifiers (e.g., calcineurin inhibitors, methotrexate, adhesion molecule antagonists)
Rheumatoid arthritis	1) Diagnosis of rheumatoid arthritis by a rheumatologist<br>2) Treatment with traditional disease-modifying antirheumatic drugs (e.g., methotrexate, sulfasalazine, hydroxychloroquine) or targeted therapy
Systemic lupus erythematosus	Diagnosis of systemic lupus erythematosus by a rheumatologist
Psoriasis	1) Diagnosis of psoriasis by a dermatologist<br>2) Treatment with corticosteroids, vitamin D analogs, anthralin, topical retinoids, calcineurin inhibitors, salicylic acid, coal tar, or moisturizers; light therapy, including sunlight, UVB phototherapy, narrowband UVB therapy, Goeckerman therapy, photochemotherapy, excimer laser, or pulsed dye laser; or systemic medications, including retinoids, methotrexate, cyclosporine, hydroxyurea, or thioguanine or targeted therapies.
Spondyloarthropathies	1) Diagnosis of ankylosing spondylitis, reactive arthritis, psoriatic arthritis, or enteropathic arthritis/spondylitis<br>2) Treatment with disease-modifying antirheumatic drugs (e.g., sulfasalazine) or targeted therapy

Supplementary Table S2. Semi-structured interview used with our participants.

**Topic**	**Questions**
**Autoimmune disease.** I would like to start this interview by asking you some questions as to how your *[AUTOIMMUNE DISEASE]* affected you at the time of making the decision on whether or not to start ICIs.	What aspects of your life were most affected by *[AUTOIMMUNE DISEASE]* at the time of making the decision on whether to start or not ICIs?
	In what ways has having cancer affected your *[AUTOIMMUNE DISEASE]*?
	What concerns did you have about the treatment of your *[AUTOIMMUNE DISEASE]*?
	What concerns or fears did you have about how your *[AUTOIMMUNE DISEASE]* might affect your cancer treatment?
**Health information needs.** Now, we are going to discuss about your understanding of your health condition at the time of making the decision on whether to start or not ICIs and your preferences for receiving health information.	Was the decision to start ICIs made by you, the doctor, or shared (by you and the doctor)? What helped to make the decision?
	What did you know about the use of ICIs for people living with cancer and *[AUTOIMMUNE DISEASE]* at the time of making the decision?
	What did you ask your doctor about ICIs before starting treatment with it?
	After receiving ICIs, did you experience any flares from your *[AUTOIMMUNE DISEASE]* or adverse events?
	Do you regret the decision made about receiving ICIs?
	What information would you have liked to have before making the decision about receiving ICIs? What other types of information about ICIs would have been good to have?
	If we wanted to send a message to all patients who are candidates to receive ICIs and have *[AUTOIMMUNE DISEASE]*, what other information do you think would be important to include to be better informed to make a decision about whether or not receive ICIs?
**Interdisciplinary communication.** We will move on to discuss the best ways and formats for learning.	What would be the best way to learn more about the benefits and the risks of ICIs for people who also have *[AUTOIMMUNE DISEASE]*?
	When would it be best to receive this information about the use of ICIs for people with cancer who also have *[AUTOIMMUNE DISEASE]*?
	Where would be the ideal place to learn about this information?
	How do you share the information about treatments that you get from other sources with your doctors?
	Do you have any ideas on how we can help others who have *[AUTOIMMUNE DISEASE],* make decisions about whether or not to take ICIs?
**Medication safety.** Now, we will discuss the concerns you had about ICIs before making the decision of whether start or not treatment.	What did you hear about or have thought about the side effects of ICIs in patients with cancer who also have *[AUTOIMMUNE DISEASE]*? Can you list some?
	What can we do to help address these side effects concerns for patients who are in the midst of making a decision about whether or not to receive ICIs?
	What potential side effect from ICIs was causing you the most concern before starting ICIs?
	After a side effect from ICIs, what information do you need to get involved in the decision-making of whether to continue or stop the ICIs?
**Preferred learning tools.** Let us now discuss how to deliver the information.<br>Maybe you can take a few moments to think back when you learned about ICIs.	What helped you to understand the information? Is there anything in particular, that helped you to better understand new information? What kind of format is better to receive health information?
	Do you have any suggestions that would make the information easy to remember?
	What is the best way to understand information about benefits and risks of ICIs, by using numbers?
	If information were to be provided about ICIs how long the information should be?
